# Patient Engagement as an Emerging Challenge for Healthcare Services: Mapping the Literature

**DOI:** 10.1155/2012/905934

**Published:** 2012-10-31

**Authors:** Serena Barello, Guendalina Graffigna, Elena Vegni

**Affiliations:** ^1^Faculty of Psychology, Università Cattolica del Sacro Cuore, Largo Gemelli 1, 20143 Milano, Italy; ^2^School of Medicine, Università degli Studi di Milano, Via di Rudinì 8, 20142 Milano, Italy

## Abstract

Patients' engagement in healthcare is at the forefront of policy and research practice and is now widely recognized as a critical ingredient for high-quality healthcare system. This study aims to analyze the current academic literature (from 2002 to 2012) about patient engagement by using bibliometric and qualitative content analyses. Extracting data from the electronic databases more likely to cover the core research publications in health issues, the number of yearly publications, the most productive countries, and the scientific discipline dealing with patient engagement were quantitatively described. Qualitative content analysis of the most cited articles was conducted to distinguish the core themes. Our data showed that patient engagement is gaining increasing attention by all the academic disciplines involved in health research with a predominance of medicine and nursing. Engaging patients is internationally recognized as a key factor in improving health service delivery and quality. Great attention is up to now paid to the clinical and organizational outcomes of engagement, whereas there is still a lack of an evidence-based theoretical foundation of the construct as well as of the organizational dimensions that foster it.

## 1. Introduction

Patient engagement is nowadays more and more recognized as a crucial component of high-quality healthcare services [1, 2]. In the majority of the Western countries, patient engagement in health and social care policies is well established with the government commit to foster interventions and research projects and methodologies which prioritize the “patient's voice” and the “patient's active roles in their own healthcare” [3, 4] as it leads to more responsive services and better outcomes of care [5]. In the last years, patient engagement has gained increasing prominence thus providing an impetus for research programs and initiatives encouraging individuals and communities to have a stronger voice in National Health Services, as it seems to contribute to gain better health outcomes, to enhance patient's care and cure experience, to improve illness self-management and adherence to therapies, and to reduce care costs.

From a nursing perspective, in particular, the need to give patients better and reliable information and more control and influence over their healthcare was particularly emphasized. By building partnership with patients and families and fostering their engagement in the process of care, nurses can develop an appropriate plan of care and cure addressing issues such as symptom and drug management and meaningful discharge goals [6]. The role of nurses constitutes a core component in the organizational aspects of care such as care coordination, continuity of care, and health information exchange: this may support the attainment of optimal, patient-centred outcomes defined by a care planning process driven by a robust partnership between nurses, physicians, and patients. Underlying these statements is the belief that engaging patients may improve patient satisfaction toward received care, cooperation and partnership with health providers, better management of the disease, increased trust, and enhanced patient-professional relationship [7]. The nursing community historically acknowledged the importance of engaging patients in the healthcare clinical encounter and in the disease management by organizing and implementing care to meet the individual's needs: however, to gain this goal, nurses have to allow patients to be engaged in their care in order to perceive their needs to be important and legitimize their expressions [8]. The consequences of nonengagement may include preventable illness and suffering, suboptimal health outcomes, increases in health disparities, and wasted resources [9].

Despite the growing popularity of the terms and the increasing attention toward this concept by researchers and policy makers, there is a little consensus about what patient engagement means. In order to address this lack of shared knowledge, this paper aimed to:

1. provide a quantitative overview of the publication trend on patient engagement from 2002 to 2012 in the whole and across different academic disciplinary fields and countries;
2. provide a qualitative analysis of the most cited academic articles in the field (the 10 most cited articles from 2002 to 2012) in order to better understand the concept of patient engagement and discuss the aspects (i.e., definition of patient engagement provided, characteristics of the study, etc.) which probably make these contribute so relevant into the scientific debate.

## 2. Materials and Methods

Analysis of the cooccurrence of the terms “patient” & “engagement” appearing in keywords, titles, and abstracts within the health academic and managerial literature was conducted on June 17st, 2012, using the electronic databases more likely to cover the core research publications in health issues (ISI Web of Science, Medline, PsychINFO, SCOPUS, Google Scholar) across medical, scientific, psychological, and social scientific sources. Together these databases allow to retrieve publications from the major academic and managerial journals across hundreds of scientific disciplines which have contributed to research on patient engagement. We decided to search articles which only included the terms “patient engagement” and not close concept (i.e., patient participation or involvement) in order to maximize sensitivity and conceptual clarity. Only research articles where the abstract was available were considered. Moreover, a study was eligible for inclusion in the analysis if in the abstract it describes (a) patient engagement generally, (b) intervention to promote patient engagement, (c) determinants of patient engagement or (d) outcomes of patient engagement, and (e) measures of patient engagement. The search was conducted within the peer-reviewed English-written literature in the last ten years for the period from January 2002 to June 2012. Articles from non-English-written journals were excluded.

We answered to aim 1 by doing bibliometric analysis on different aspects of publications' trend; aim 2 was addressed by conducting a qualitative content analysis on some selected articles.

- Bibliometric analysis of the articles retrieved from all the databases were performed in order to portrait the trend of published articles within the academic and managerial fields.
- A deeper quantitative bibliometric analysis was conducted on the mere academic production subcorpus (i.e., articles from peer review journals with IF) in order to detect disciplines and countries more “productive” in the ongoing debate about patient engagement. In this case, we chose to analyze only the research articles indexed in the Scopus database as it provides the wider amount of publications (in comparison to the other academic databases)—see Table 1—and allows to cover all different disciplines involved in the health research, whereas Medline and PsychInfo are more discipline based [20, 21]. (In comparison to Isi Web of Science, Scopus has almost 28 million records against the 19 million of Isi Web of Science and covers over 15,000 journals versus 9,000 in Isi Web of Science [22]. Scopus allow to extract the academic field more responsive to patient engagement issues by mapping and labeling the articles under consideration basing on the judgment of a pull of expert in the health sciences. The specific academic field which each article belongs to was based on both Scopus pull of experts' judgment and a manual qualitative revision of the abstract of the ambiguous ones by one of the author (SB). The “create-citation report” tool was used to obtain detailed data on citations to the retrieved publication).
- Finally, the search results were exported in text format: this allowed one of the authors (SB) to analyze in depth the 10 most cited articles (themes and features) and to conduct a qualitative content analysis [23].

## 3. Results

### 3.1. Bibliometric Analysis

Descriptive bibliometric analysis of retrieved data was performed to analyze the quantitative trend of publications about patient engagement—over the 10-year period considered—taking into account the number of academic and managerial articles provided by all the databases. Data showed a general increasing interest toward patient engagement both in the academic and managerial fields. This is clearly shown by the growing trend of published items over the considered period (Table 1). The number of publications per years indexed by all the used databases clearly highlights a progressive increase in the yearly number of publication related to this area.

The academic fields—in terms of article-related contents—that are more involved in publishing on patient engagement are medicine, which covers the 69, 1% of the entire corpus of publication on this theme, followed, in percentage, by nursing (16, 2%). Psychology and social science academic production follow with the 9, 6% and the 5, 1% of the total amount of publications, respectively. Furthermore, the trend of publication across disciplines per years displays a general growing interest around the topic in particular in the medical and nursing literature; in contrast, the contribute of the psychological research appears as decreasing from 2004 up today. For more detailed data, see Table 2.

**Figure 1:**
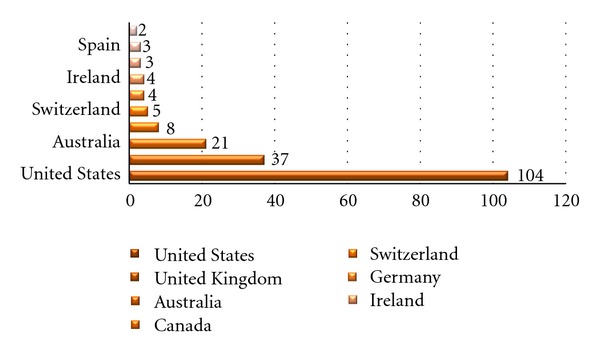
Number of publications by country first authors' affiliation.

Focusing on the academic articles provided by Scopus, the number of publications by countries, considering the first author's affiliation, highlights the US predominance in publishing about patient engagement related topics with an amount of 104 publications (48, 2%) in ten years, thus contributing around to the half of all publications about patient engagement listed in Scopus. Altogether, academic production of authors belonging to other countries covers the remaining amount of publications. Further details about the number of publications by country can be found in Figure 1.

### 3.2. Qualitative Content Analysis

Qualitative content analysis of the abstract of the ten most cited articles published from 2002 to 2012 was conducted in order to give a general overview of the most common research topics related to patient engagement and to provide a preliminary suggestion about the underlying dimensions of patient engagement thus aiming to lay the foundation for a shared definition (see Table 3).

Patient engagement appears as a fragmented concept lacking of a unique definition: in some papers it is described as a set of healthy behaviors which individuals should perform in terms of adherence to drug and therapeutic prescriptions [10, 16]: patient engagement, in this sense, constitutes a measurable behavioral marker of patients' compliance to therapies and their ability in managing symptoms. Other authors described engagement as a cognitive (i.e., knowledge and illness believes) or a relational (i.e., the quality of patient-clinician encounters) factor which influences patient's emotional experience with healthcare delivery and fosters patient alliance with clinicians [11–13, 15, 17, 18]. Finally, in some contributes, patient engagement is considered more generally as an organizational feature that constitutes a crucial element in health policy making to deliver effective and high-quality healthcare interventions as it seems to reduce waste of resources, health service abuse, and improve health outcomes [14, 19].

## 4. Discussion

The increasing attention to patient engagement and related topics is clearly shown by the growing number of publications from 2002 to 2012 thus suggesting that empowering patients to take an active role and to be engaged in their care has been internationally identified as a key factor in the drives to improve health service delivery and quality [24]. Moreover, it is interesting to note that all the academic communities involved in health research share an interest in studying patient engagement as a core condition in performing effective chronic illness coping and management [25]. These data are relevant as they foster the need to critically assess the specific application of patient engagement to the health services specificities. The predominance in the number of publications within the medical and nursing areas may suggest that these fields are probably the most responsive to the debate on patient engagement by including it in the research agenda and, at the same time, by producing insights in order to implement change in healthcare organization processes [26, 27]. Regarding the trend of publications split by discipline, even though there is a general increasing of academic production over the years, in the period from 2002 to 2005, we can observe a prominent focalization on the mental health context which implies a conceptualization of engagement as alliance between patient and clinicians as a key factor in promoting treatment effectiveness. In the last years, from 2006 to 2012, a more specific focus on organic patients' care (by medical and nursing perspectives) provided evidence about the need for developing interventions to improve disease-specific self-management behaviors, such as medication, adherence, and condition monitoring in order to better allocate resources to manage the whole patient population. This shift in the ways of conceptualizing patient engagement does not facilitate the formulation of a shared definition across scientific communities thus supporting the idea that a comprehensive definition is a challenging but urgent task. Furthermore, the efforts in encouraging greater patient engagement seem to be founded, up to now, on research more focused on expected pragmatic impact on patients' health obtained by active partnering with them and not on the organizational process which sustains its achievement: as a consequence, the current academic debate seems to reveal a stronger interest in the clinical and organizational outcomes of patient engagement (may be due to the need for legitimizing it as a healthcare priority) [12, 19]. However, little importance is till now given to cast light on the intrinsic nature of engagement: despite the growing popularity of the term patient engagement in the everyday rhetoric of worldwide NHS—especially in the English-speaking countries—it remains conceptually underdeveloped. Few attempts to find empirical markers of engagement have been conducted [9, 28], probably due to the still fragile link between the empirical evidence and the theoretical foundation of this construct. As a consequence, in order to develop a robust evidence-based theoretical framework and to enable data comparison and evaluation to be made, there is the need for a common understanding of what is meant by patient engagement in practice and how it can be operationalized and measured.

The multifaced definition of patient engagement emerging from our data suggests the hypothesis for which this concept may have some unchanging and underlying dimensions that are discipline-unrelated and operationalizations that are, instead, idiographic and context based. Probably, patient engagement may be observed from multiple perspective thus suggesting the opportunity to reflect upon the interaction between its individual (i.e., emotional, cognitive and behavioral, etc.), relational (i.e., patient-health providers, patient-caregiver, patient-patient, etc.), and organizational (i.e., type of healthcare settings, admission process, shape and process of intervention, use of ICT, role and attitude of health professionals, etc.) dimensions across the specificities of each single disease. A recovery of a psychological view seems also to be necessary, in order to give a comprehensive theorization which may take into account the individuals' role in being engaged in their care as subjects involved into a relational context and into a specific health culture: this may lead to build an inductively founded theoretical framework based on empirically rooted data. Surely, to take into account the patients' perspective on their own engagement may be particularly relevant in order to better assess the full range of factors that may be involved in such engagement and to foster a more effective use of healthcare services [27]. Given the complexity of the phenomenon and the relevance of its practical implications, our study may suggest that there is a pressing need for empirical research to deepen the components of patient engagement at various levels (individual, relational, organizational, etc.), their specific impact and their interconnection [28]. Once these issues are addressed, targeted interventions could be developed and implemented.

On the other hand, the contributions of nursing research in valuing the central role of nursing practice in enhancing patient's engagement in the process of care should be encouraged. Nurses are often portrayed by patients as the health providers who most make them feel as full engaged partners in the process of care [29]. The input of nursing research in highlighting factors which may shape the possible forms of patients participation really attuned to each patient's needs may offer useful insights to plan interventions which maximize opportunities for patient's to take an active part in their care, if they so whish. 

In order to gain these aims, we think that to assemble a detailed picture of the underlying components of patient engagement is particularly urgent: this may allow to offer a holistic vision on patient engagement which takes into account its multidimensional nature which could better enable strategic tailoring and targeting of interventions to support the capacity of health organization to be “engager” and to make patients “engaged” in the care process. Finally, engaging patients in interventions which develop their skills and confidence in self-management might be a key strategy in order to address the necessity of policy makers for seeking ways to rationalize the use of resources in an effort to deal more effectively with long-term chronic conditions and disabilities [30]. The growing claim for patients that are fully engaged and mobilized should be not only a declaration of intent, but also a strategic resource which could transform the quality and sustainability of health system [31]. The implications of this study are likely to have relevance for international patient engagement intervention and to gain insights into its related body of knowledge in order to open viable avenues for further research.

### 4.1. Limits and Future Research Developments

This bibliometric exploration offered an interesting picture of the ongoing debate about patient engagement. However, further analysis is needed in order to deepen the definition of patient engagement and to systematically meta-analyze results achieved on the topics by scholars across disciplines [32]. A deeper consideration of pathological areas and of how these impact on the different conceptualizations of patient engagement is also opportune. Moreover, empirical research is needed in order to ecologically explore what means for patients to feel engaged in the care and cure process and to collect stories and experiences of healthcare intervention fruitions that appeared successful in improving their engagement.

## Figures and Tables

**Table 1:** Number of publications across years and databases.

Year	Electronic databases					Total	
	Medline	Psychinfo	Scopus	ISI Web of science	Google scholar	*N *	%
	*N *	*N *	*N *	*N *	*N *		
2002	4	4	3	2	61	74	1,7
2003	6	4	5	5	102	122	2,9
2004	8	3	5	5	87	108	2,5
2005	10	7	9	6	121	153	3,6
2006	11	9	11	8	176	215	5,1
2007	16	8	19	13	244	282	6,7
2008	27	14	23	19	316	399	9,4
2009	27	11	17	13	445	513	12,2
2010	43	19	47	39	652	800	19
2011	66	23	46	40	779	954	22,7
2012*	[ *56*]	[ *6*]	[ *32*]	[ *26*]	[ *479*]	[ *599*]	[ *14,2*]

Total	162	96	185	150	3261	3620	100
	[*218*]	[*102*]	[*217*]	[*176*]	[*3740*]	[*4219*]	

*2012 is reported but not considered to discuss the findings as data are related only to the period from January 2012 to June 2012. The amount of publications including 2012 is reported in square brackets.

**Table 2:** Number of publications by year and by discipline.

Discipline	Number of publications per year											Total
	2002	2003	2004	2005	2006	2007	2008	2009	2010	2011	2012*	*N*
Medicine	3	4	5	6	8	12	14	13	24	39	[* 29*]	128 [* 150*]
Nursing	2	1	1	2	2	3	3	5	6	4	[ *6*]	29 [* 35*]
Psychology	0	5	5	2	2	1	1	0	1	1	[* 1*]	20 [* 21*]
Social science/health policies	0	2	1	2	2	4	4	2	2	8	[* 3*]	8 [* 11*]

*2012 is reported but not considered to discuss the findings as data are related only to the period from January 2012 to June 2012. The amount of contributions including 2012 is reported in square brackets.

**Table 3:** Ten most cited publications from 2002 to 2012 (*the double labeling is due to the discipline label assigned by Scopus reviewers).

Number of citations	Author(s)	Title	Year	Source	Discipline*	Definition of patient engagement	Reference
188	Lehman et al.	Assessing organizational readiness for change	2002	Journal of Substance Abuse Treatment	Medicine	Engagement as *actions* individuals perform in terms of *adherence to drug prescription* and a key component *for high-quality *healthcare services	[10]

120	Simpson	A conceptual framework for drug treatment process and outcomes	2004	Journal of Substance Abuse Treatment	Medicine	Engagement as a factor which enables *patient alliance* with clinicians and *enhance recovery experience *	[11]

119	Davis et al.	A 2020 vision of patient-centered primary care	2005	Journal of General Internal Medicine	Medicine	Engagement as *a key component* to foster *patient-centred medical approach *	[12]

73	Hibbard et al.	Do increases in patient activation result in improved self-management behaviors?	2007	Health services research	Nursing/social science	Engagement as a *behavioural activation* related to healthy behaviours and positive health outcomes	[13]

49	Roy-Byrne and Wagner	Primary care perspectives on generalized anxiety disorder	2004	Journal of Clinical Psychiatry	Medicine/psychology	Engagement as a *crucial elements in * *health policy making* to deliver effective and high-quality healthcare interventions	[14]

42	Casale et al.	ProvenCareSM: A provider-driven pay-for-performance program for acute episodic cardiac surgical care	2007	Annals of surgery	Medicine	Engagement as *behavioral activation* that contributes to reduce resource abuse and improve health outcomes	[15]

41	Trotti et al.	Patient-reported outcomes and the evolution of adverse event reporting in oncology	2007	Journal of Clinical Oncology	Medicine	Engagement as a *measurable marker of patients' compliance* to therapies and *symptoms' management *	[16]

32	Franklin et al.	Patients' engagement with “Sweet Talk”—a text messaging support system for young people with diabetes	2008	Journal of Medical Internet Research	Medicine	Engagement as a *cognitive, behavioural, emotional, and social construct* which foster patient's self-management	[17]

31	McCracken	Social context and acceptance of chronic pain: the role of solicitous and punishing responses	2005	Pain	Medicine/psychology	Engagement as a *behavioural activation* useful to better control and manage illness symptoms and emotional-related alterations	[18]

30	Villagra	Strategies to control costs and quality: a focus on outcomes research for disease management	2004	Medical care	Nursing/social science	Engagement as a *measurable marker of clinical results* and organizational factor which contributes to *reduce healthcare * *costs *	[19]

## References

[1] Coulter A (2002). *The Autonomous Patient: Ending Paternalism in Medical Care*.

[2] Forbat L, Cayless S, Knighting K, Cornwell J, Kearney N (2009). Engaging patients in health care: an empirical study of the role of engagement on attitudes and action. *Patient Education and Counseling*.

[3] Mockford C, Staniszewska S, Griffiths F, Herron-Marxs S (2011). The impact of patient and public involvement in UK NHS health care: a systematic review. *International Journal for Quality in Health Care Advance*.

[4] Graffigna G, Vegni E, Barello S, Olson K, Bosio CA (2011). Studying the social construction of cancer-related fatigue experience: the heuristic value of Ethnoscience. *Patient Education and Counseling*.

[5] Executive NHS (1999). *Patient and Public Involvement in the New NHS*.

[6] Grant B, Colello S (2009). Engaging the patient in handoff communication at the bedside. *Nursing*.

[7] Cahill JO (1998). Patient participation—a review of the literature. *Journal of Clinical Nursing*.

[8] Morse JM, Bottorff J, Anderson G, O’Brien B, Solberg S (2006). Beyond empathy: expanding expressions of caring. *Journal of Advanced Nursing*.

[9] Gruman J, Rovner MH, French ME (2010). From patient education to patient engagement: implications for the field of patient education. *Patient Education and Counseling*.

[10] Lehman WEK, Greener JM, Simpson DD (2002). Assessing organizational readiness for change. *Journal of Substance Abuse Treatment*.

[11] Simpson DD (2004). A conceptual framework for drug treatment process and outcomes. *Journal of Substance Abuse Treatment*.

[12] Davis K, Schoenbaum SC, Audet AM (2005). A 2020 vision of patient-centered primary care. *Journal of General Internal Medicine*.

[13] Hibbard JH, Mahoney ER, Stock R, Tusler M (2007). Do increases in patient activation result in improved self-management behaviors?. *Health Services Research*.

[14] Roy-Byrne PP, Wagner A (2004). Primary care perspectives on generalized anxiety disorder. *Journal of Clinical Psychiatry*.

[15] Casale AS, Paulus RA, Selna MJ (2007). “ProvenCareSM”: a provider-driven pay-for-performance program for acute episodic cardiac surgical care. *Annals of Surgery*.

[16] Trotti A, Colevas AD, Setser A, Basch E (2007). Patient-reported outcomes and the evolution of adverse event reporting in oncology. *Journal of Clinical Oncology*.

[17] Franklin VL, Greene A, Waller A, Greene SA, Pagliari C (2008). Patients’ engagement with “Sweet Talk”—a text messaging support system for young people with diabetes.. *Journal of Medical Internet Research*.

[18] McCracken LM (2005). Social context and acceptance of chronic pain: the role of solicitous and punishing responses. *Pain*.

[19] Villagra V (2004). Strategies to control costs and quality: a focus on outcomes research for disease management.. *Medical care*.

[20] Jacso P (2005). As we may search—comparison of major features of the Web of Science, Scopus, and Google Scholar citation-based and citation-enhanced databases. *Current Science*.

[21] Burnham JF (2006). Scopus database: a review. *Biomedical Digital Libraries*.

[22] Bakkalbasi N, Bauer K, Glover J, Wang L (2006). Three options for citation tracking: Google Scholar, Scopus and Web of Science. *Biomedical Digital Libraries*.

[23] Morse JM, Field PA (1995). *Qualitative Research Methods for Health Professionals*.

[24] Crawford MJ, Rutter D, Manley C (2002). Systematic review of involving patients in the planning and development of health care. *British Medical Journal*.

[25] Coulter A, Rozansky D (2004). Full engagement in health. *British Medical Journal*.

[26] Hogg CNL (2007). Patient and public involvement: what next for the NHS?. *Health Expectations*.

[27] Barello S, Graffigna G, Vegni E Conceptualizing patient engagement in healthcare: a thematic software-based analysis.

[28] Nolan M, Caldock K (1996). Assessment: identifying the barriers to good practice. *Health and Social Care in the Community*.

[29] Hibbard JH, Stockard J, Mahoney ER, Tusler M (2004). Development of the patient activation measure (PAM): conceptualizing and measuring activation in patients and consumers. *Health Services Research*.

[30] Grol R, Wensing M, Grol R, Wensing M, Eccles M (2005). Effective implementation: a model. *Improving Patient Care: The Implementation of Change in Clinical Practice*.

[31] Schoen C, Osborn R, How SKH, Doty MM, Peugh J (2009). In chronic condition: experiences of patients with complex health care needs, in eight countries, 2008. *Health Affairs*.

[32] Barello S, Vegni E, Graffigna G, Graffign G, Morse JM, Bosio AC Conceptualizing patient engagement in healthcare: a thematic software-based analysis.

